# Extensive protein hydrolyzation is indispensable to prevent IgE-mediated poultry allergen recognition in dogs and cats

**DOI:** 10.1186/s12917-017-1183-4

**Published:** 2017-08-17

**Authors:** Thierry Olivry, Jennifer Bexley, Isabelle Mougeot

**Affiliations:** 1 Department of Clinical Sciences, College of Veterinary Medicine, North Carolina State University, Raleigh, NC USA; 2 Comparative Medicine Institute, College of Veterinary Medicine, North Carolina State University, Raleigh, NC USA; 3 Avacta Animal Health, Leeds, Wetherby UK; 4 Royal Canin, Aimargues, France

**Keywords:** Allergy, Canine, Food, Feline, Hydrolysate, Hypersensitivity, IgE

## Abstract

**Background:**

The central premise for the commercialization of diets with hydrolyzed ingredients is that the small-sized digested peptides would be unable to crosslink allergen-specific IgE at the surface of tissue mast cells and induce their degranulation. Evidence for the validity of this concept to diagnose food allergies in dogs and cats is limited, however. Our objectives were to study the recognition of standard and variably hydrolyzed poultry extracts by sera from dogs and cats with elevated chicken-specific serum IgE.

**Results:**

Forty sera from dogs and 40 from cats with undetectable, low, medium or high serum levels of chicken-specific IgE were tested by ELISA on plates coated with the positive controls chicken, duck and turkey meat extracts and the negative controls beef meat (dogs) or wheat (cats). Plates were also coated with a non-hydrolyzed chicken meal, and mildly- or extensively-hydrolyzed poultry feather extracts. The frequencies of dogs with positive IgE against the various extracts were: chicken meat: 100%, duck and turkey meats: 97%, beef meat: 3%, non-hydrolyzed chicken meal: 73%, mildly-hydrolyzed poultry feathers: 37% and extensively-hydrolyzed poultry feathers: 0%. For cats, these respective percentages were (with wheat replacing beef as a negative control): 100, 84, 97, 7, 7, 0 and 0%. To detect any allergenic cross-reactivity between poultry meat-based and feather hydrolysate-derived extracts, an IgE ELISA inhibition was also done. Ten canine sera with the highest level of anti-poultry IgE in the previous experiment were incubated overnight with a previously optimized 50 μg amount of each of the extracts used above. We performed ELISA on plates coated with chicken, duck or turkey meats with or without inhibitors. The median inhibition percentages after incubation with the non-hydrolyzed chicken meal were ~22%, with the mildly-hydrolyzed poultry feathers: 14–22%, and those with the extensively-hydrolyzed poultry feathers: 5 to 10%; the last inhibition level was similar to that of the beef meat negative control.

**Conclusions:**

Altogether, these results suggest that an extensive—but not partial—hydrolyzation of the poultry feather extract is necessary to prevent the recognition of allergenic epitopes by poultry-specific IgE.

**Electronic supplementary material:**

The online version of this article (doi:10.1186/s12917-017-1183-4) contains supplementary material, which is available to authorized users.

## Background

In dogs and cats, as in humans, the diagnosis of adverse food reactions (AFRs) relies on the recurrence of clinical signs after provocation with causative food ingredients. Such food re-challenges are typically done after a lengthy period of dietary restriction (i.e. “elimination”) to permit the disappearance of existing clinical signs and the return to a state of normalcy that allows the detection of positive provocations [1]. In companion animals, restrictive dietary trials usually consist of feeding ingredients not eaten previously [1] for 6 to 8 weeks [2].

Finding a diet with “novel” ingredients, be it home-cooked or commercially available, is becoming increasingly challenging because of the diverse—often unusual and sometimes even “exotic”—regimes eaten by today’s pets and the rising complexity of currently available commercial foods. Furthermore, there is evidence that some commercial diets are contaminated by ingredients not listed in their composition label [3–7]. Finally, the characterization of some food allergens for dogs [8, 9], as well as our recent serological study [10], revealed the existence of extensive IgE cross-reactivity among allergens of taxonomically-related food groups in this species. Such studies suggest that some of these “novel” ingredients might cross-react with historically fed components, unless the protein source were evolutionarily distant from all those eaten previously. Altogether, the points raised above should reduce our confidence in being able to perform “accurately-restricted” dietary trials using so-called “novel” food items.

To alleviate the need for a perpetual quest to identify new ingredients and to reduce problems of allergenic cross-reactivity, pet food manufacturers created diets containing proteins hydrolyzed into peptides of a size supposedly insufficient to induce IgE-mediated mast cell activation (reviewed in [1]). For many years, and due to production costs and technical aspects, only partially hydrolyzed diets were commercialized. While these hydrolysate-based petfoods clearly exhibited a reduced allergenicity, there was evidence that some dogs with cutaneous AFRs still reacted clinically to their ingestion, thereby suggesting that some proteins were insufficiently digested [11, 12]. The development of Royal Canin Anallergenic (RCA, also known in North America as Ultamino, Royal Canin, Aimargues, France) was noteworthy, not only because of its high-grade of protein hydrolyzation resulting in a diet almost entirely composed of single amino acids and very short peptides, but also because of its protein source (poultry feathers) being potentially quite different in protein composition from that of other meat-based poultry-based diets. A recent study established the relevance of extensive protein hydrolyzation to reduce clinical allergenicity, as ten chicken-allergic dogs did not react clinically after two weeks exclusively eating the poultry feather-containing RCA [12].

To further study if protein hydrolyzation results in reduced immunological allergenicity in companion animals, as it does for food allergic humans, we set out to investigate if partially- or extensively-hydrolyzed poultry feather extracts were recognized by serum IgE from dogs and cats sensitized to poultry meats. We will demonstrate herein that only the extensive hydrolyzation of the poultry feather extract results in a lack of IgE recognition by poultry-specific IgE, thereby confirming the validity of extensive hydrolyzation for hypoallergenic pet food development.

## Methods

### Animal sera

Surplus canine and feline sera submitted to Avacta Animal Health (Wetherby, Leeds, UK) by veterinarians for food allergen-specific IgE serological testing were used for this study. For dogs, the panel consisted of beef, pork, lamb, duck, chicken, turkey, wheat, soybean, barley, rice, potato, corn, oat, cow’s milk, whole hen’s egg, white fish, venison, salmon and rabbit. For cats, the panel included beef, pork, lamb, duck, chicken, turkey, rabbit, salmon, tuna, white fish, wheat, soybean, rice, corn, cow’s milk and whole hen’s egg. All dog and cat sera had been stored at −80 °C for a maximum of two and six years, respectively. Canine sera were selected according to their original levels of serum chicken-specific IgE (IgE reactivities expressed as optical density, OD, values at 405 nm, OD_405_ uncorrected for background) in the Sensitest commercially available ELISA (Avacta).

The D1 group (30 dogs) contained the following sera:

- D1-LCR: ten dogs with low chicken IgE reactivity and a previous OD_405_ between 0.3 and 0.4 in the Sensitest assay.
- D1-MCR: ten dogs with moderate IgE chicken reactivity (OD_405_ between 0.5 and 0.9)
- D1-HCR: ten dogs with high chicken IgE reactivity (OD_=405_ > 1.0)

In their previous ELISAs done at the time of the original submission, all of the dogs in this group had detectable IgE to at least one food extract; one dog was only positive to chicken and none had any detectable IgE reactivity to beef.

D2-NCR: This group included sera from ten dogs with undetectable levels of IgE to chicken (i.e. “non-chicken reactive dogs” NCR; OD_405_ < 0.3). By previous ELISAs, these dogs had no visible IgE reactivity to any food tested on the panel.

Similarly, we selected feline sera based upon their previously determined levels of serum chicken-specific IgE (OD_405_, uncorrected for background).

The C1 group was composed of 31 cats divided into the following subgroups:

- C1-LCR: eleven cats with low chicken IgE reactivity (OD_405_: ~0.2–0.4)
- C1-MCR: ten cats with moderate chicken IgE reactivity (OD_405_: ~0.5–1.0)
- C1-HCR: ten cats with high chicken IgE reactivity (OD_=405_: >1.0)

By previous ELISAs, all of the cats in this group had at detectable IgE to at least three foods. All cats had measurable IgE against beef but none had IgE reactivity to wheat.

Finally, the C2-NCR group comprised sera from nine cats with undetectable levels of IgE to chicken in the Sensitest ELISA (OD_405_: <0.2). By previous ELISAs, there was no IgE reactivity to any food in these cats.

Details from all selected canine and feline sera are found in the Additional file 1.

### Extracts

Raw chicken meat (CMT), turkey meat (TMT), duck meat (DMT), beef meat (BMT) and wheat (WHT) extracts were purchased from Greer Laboratories (Lenoir, NC, USA). Non-hydrolysed chicken meal (NHCM, the ground, rendered clean parts of the carcasses of slaughtered poultry), mildly-hydrolysed poultry feathers (MHPF) and extensively-hydrolysed poultry feathers (EHPF) were obtained from Royal Canin. The main difference between the MHPF and EHPF was the presence of residual proteins of molecular weight superior to 10 kDa in the former, but not in the latter; the EHPF were those used in the RCA. The commercial-grade material was defatted in acetone, filtered and then dried. Allergens were obtained after two series of successive extractions in phosphate-buffered saline (PBS), followed by centrifugation and, ultimately, by dialyzing the supernatant against distilled water. The protein concentration of each extract was determined at 280 nm by Trinean DropSense96 (Trinean, Gent, Belgium). The final protein concentrations of the NHCM, MHPF and EHPF extracts were 6.8, 3.1 and 2.4 mg/ml, respectively. Extracts were stored at 4 °C before use.

### ELISAs

Canine and feline food allergen-specific IgE serum levels were determined by ELISA. Microtiter plates (Costar, Corning, NY, USA) were coated overnight at 2–8 °C with 50 μL/well of each extract, diluted to a concentration of 5 μg/mL of protein in 0.05 M carbonate/bicarbonate buffer, pH 9.6.

Plates were washed three times with 150 μL/well Tris-buffered saline (TBS) with 0.05% Tween-20 (TBST) and blocked with 150 μL/well TBS containing 0.5% sucrose and 0.5% PVP10 (block) for 2 h at room temperature. After removal of the block, plates were dried at 37 °C for 2 h. Canine and feline sera were diluted 1:10 in TBST, and incubated with coated extracts overnight at 2–8 °C (50 μL/well. For standardization of the canine and feline ELISAs, serial three-fold dilutions of pools of dog and cat reference sera with high levels of anti-beef IgE (for dog tests) or chicken (for cat tests) were included on each plate. Undiluted, these two standard pools were assigned a value of 500 arbitrary units (AU). Positive and negative serum controls, with moderate and negligible levels of beef- (dog tests) or wheat- (cat tests) specific IgE, respectively, were also diluted at 1:10 in TBST and included on each plate. Plates were washed with TBST, as before, and incubated with 0.5 μg/mL alkaline phosphatase-labelled anti-dog IgE (clone 5.91; Bruce Hammerberg, NC State University, Raleigh, NC, USA) for 2 h at RT. This mouse monoclonal antibody has been described previously [13] and shown to recognize IgE from several mammalian species including cats [14]. The specificity of this antibody for dog IgE was confirmed in previous ELISAs, as a strong signal (OD_405_: 1.8) was found on a 0.5 μg/mL dog IgE coat without any concurrent detection (OD_405_: 0.11) of dog IgG coated at up to 20 μg/mL. After the final three washes with TBST, plates were developed with 50 μL/well of an alkaline phosphatase substrate (pNPP; BioFx Laboratories, Owings Mills, MD, USA) for 30 min at room temperature. The reactions were stopped by the addition of 50 μL/well 1 M NaOH, and OD_405_ were determined using a microplate reader (Tecan, Männedorf, Switzerland). For both ELISAs, standard curves were generated by fitting the mean standard uncorrected OD_405_ to a sigmoidal 4PL curve (Prism, Graphpad Software, La Jolla, CA, USA) with log10 concentrations of the standard dog or cat serum pools.

### Inhibition ELISAs

Inhibition ELISAs were performed to determine if NHCM, MHPF and EHPF extracts could block serum IgE binding to poultry extracts, thereby demonstrating the presence of shared IgE binding epitopes. Ten dog sera that showed the highest IgE reactivity to CMT, DMT and TMT in the ELISAs described above (i.e. the HCR sera) were selected for the inhibition assays. Due to insufficient volumes available, inhibitions were not performed with the feline sera. Inhibitor solutions of NHCM, MHPF and EHPF were prepared in TBST, each to a concentration of 500, 1000 and 2000 μg/mL (i.e. 100×, 200×, and 400× coat level, respectively). To serve as the positive inhibitor, the CMT, DMT and TMT were combined, as a three-meat extract in a 1:1:1 ratio of equal protein levels, to the same concentrations as that of the extracts above. As a negative inhibitor, we diluted the BMT extract to the same concentrations. Dog sera were pre-incubated overnight at 2–8 °C, at a dilution of 1:10, with 50 μL each of the three increasing concentrations of the test and control inhibitor solutions. Additionally, each dog serum was diluted at 1:10 in TBST in the absence of inhibitor. All serum-inhibitor mixes and no-inhibitor controls were then tested at 50 μL/well by ELISA on the CMT, DMT and TMT extracts, as described above. For any given concentration of inhibitor, the percentage inhibition was calculated as follows: 100 – ((OD405 of serum with inhibitor/OD405 of serum without inhibitor) ×100) where OD405 represents the background-corrected absorbance at 405 nm.

### Immunoblotting

The extracts above, thawed after two years of freezing, were heated at 70 °C for 10 min in the presence of 5% Criterion XT reducing agent (Bio-Rad Laboratories, Hercules, CA, USA) before sodium dodecyl sulfate polyacrylamide gel electrophoresis (SDS-PAGE). Proteins (5 μg per lane) were separated on 4–12% Bis-Tris Criterion XT precast gels (Bio-Rad) at 180 V using MES (4-morpholineethane-sulfonic acid) running buffer on a Criterion Cell system (Bio-Rad). Molecular weight markers (10–190 kDa; PageRuler Plus, Thermo Scientific) were run in parallel to the extracts. Following electrophoresis, the gels were either stained using InstantBlue Coomassie stain (Sigma Aldrich, Dorset, UK) to verify protein loading and proteins were transferred to a polyvinylidene difluoride (PVDF) membrane (BioRad). This was performed at 100 V for 1 h in Tris/glycine transfer buffer (Bio-Rad) containing 20% methanol using Criterion Blotter apparatus (Bio-Rad). The membrane was blocked with TBS containing 0.5% PVP10 at room temperature for 2 h, and then incubated with a serum pool from three dogs with high chicken-specific IgE levels (>OD_405_: 1.0, by previous ELISAs), diluted at 1/10 in TBST. The membrane was incubated with serum at RT overnight. After washing three times with TBST, the membrane was incubated with alkaline phosphatase-conjugated anti-dog IgE antibody (clone 5.91, 0.5 μg/ml in TBST) at room temperature for 2 h with shaking. The membrane was washed three times with TBST and rinsed twice prior to addition of nitroblue tetrazolium/5-bromo-4-chloro-3-indolyl-phosphate substrate (Thermo Scientific). The reaction was stopped with deionised water and blots were then dried at room temperature. Images of gels and blots were captured using a G:BOX Chemi-XR5 gel imaging system (Syngene, Cambridge, UK).

### Statistics

Data were analyzed with SAS v9.3 (SAS, Cary, NC, USA). The frequencies of canine or feline positive ELISA tests with the various extracts were compared with permutation tests-based on Fisher’s exact test with a resampling size of 1000 times. Then, the alpha inflation risk subsequent to multiple extract comparisons was controlled with false discovery rate adjustments. The level of statistical significance was set at*P* < 0.05, for two-sided analyses.

## Results

### ELISA validation

The canine ELISA standard curve had an accuracy of between 99 and 117% at concentrations varying between 3.7 and 100 AU. The inter-assay coefficients of variation (made from 14 measurements over seven plates) of the negative and positive controls were 12 and 7%, respectively. We established the positive threshold of 8.1 AU as the mean OD_405_ plus three standard deviations of a large number (n = 95) of canine sera without detectable anti-poultry IgE.

For the feline ELISA, the standard curve was 99–106% accurate over the same concentration range as above. The inter-assay coefficient of variation of the positive control was 6%. The positive threshold—determined as for the canine ELISA, but with a similar number and type of feline sera—was calculated to be 6.2 AU.

### Canine ELISAs

**Fig. 1:**
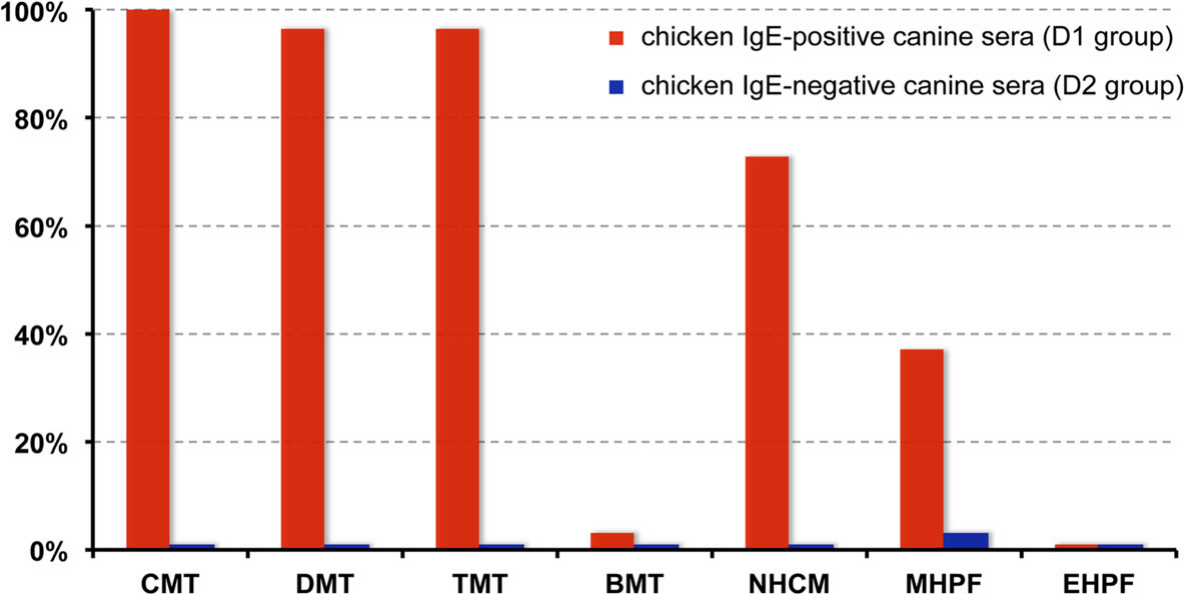
Frequencies of positive canine sera tested on the various extracts

The frequencies of group D1 and D2 sera testing positive on the various extracts are shown in Fig. 1. Among the 30 dogs from the D1 group, the number (and frequencies) of dogs testing positive for the CMT, DMT and TMT extracts were 30 (100%), 29 (97%) and 29 (97%) respectively. All dogs that had positive reactions to TMT were also positive to DMT and CMT; all sera positive to DMT were also positive to TMT and CMT. Only one serum (3%) was positive to the BMT extract.

Twenty-two sera (73%) were positive with the NHCM, and these were also positive for the CMT. When tested on the two hydrolysates, 11 (37%) and no dogs (0%) reacted to the MHPF and EHPF extracts, respectively; sera positive on the MHPF were also positive when tested on the CMT and NHCM extracts. Details on the actual frequencies of reactivity of the various D1 serum subgroups are provided in the Additional file 2. As expected, sera from the D1-LCR subgroup had the lowest rate of positive tests among those of the D1 group.

The proportions of positive canine sera tested on the various extracts were compared statistically and results are shown in Table 1. Of note is that the proportion of reactivity to the EHPF extract was significantly lower than those to all other extracts, except for the BMT negative control.

**Table 1:** statistical comparisons between frequencies of positive canine sera tested on the various food extracts

	CMT	DMT	TMT	BMT	NHCM	MHPF	EHPF
CMT	-	ns	ns	***	ns	***	***
DMT		-	ns	***	ns	***	***
TMT			-	***	ns	***	***
BMT				-	***	*	ns
NHCM					-	ns	***
MHPF						-	**

*ns* not significant; *P < 0.05; **P < 0.01; ***P < 0.001

In the group D2 (NCR), nine of ten sera (90%) had negative results for all tested extracts; one (10%) was a very weak positive (8.8 AU; positive threshold: 8.1) when tested on the MHPF extract.

### Feline ELISAs

**Fig. 2:**
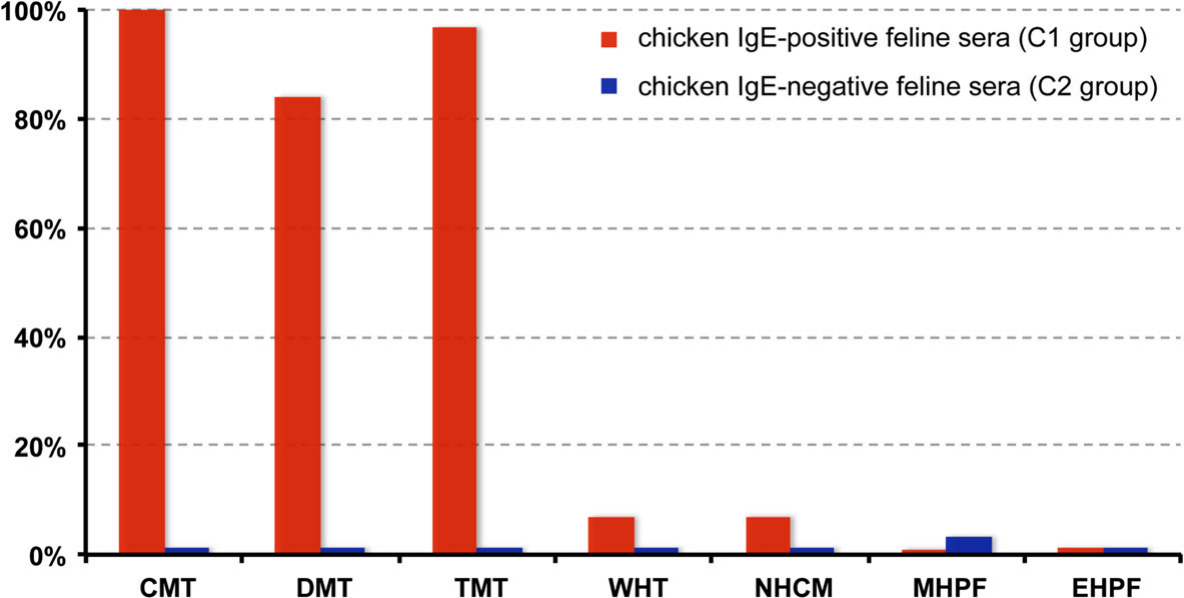
Frequencies of positive feline sera tested on the various extracts

All frequencies of positive tests using C1 and C2 sera and the various extracts are depicted in Fig. 2.

All 31 feline sera from the C1 group tested positive on the CMT; 30 (97%) reacted positively to TMT and 26 (84%) to the DMT. Two sera (7%) had a positive test on the NHCM extract, and these were also positive for the CMT, DMT and TMT; two reacted to the WHT extract (7%). In contrast, none of the sera reacted to either the MHPF or the EHPF extracts. The proportions of positive sera within the three C1 serum subgroups are available in the Additional file 2.

The proportions of C1 sera testing positive to CMT, DMT, and TMT extracts were not significantly different from each other (Table 2). Similarly, these percentages were not significantly different between WHT, NHCM, MHPF and EHPF.

**Table 2:** statistical comparisons between frequencies of positive feline sera tested on the various food extracts

	CMT	DMT	TMT	WHT	NHCM	MHPF	EHPF
CMT	-	ns	ns	***	***	***	***
DMT		-	ns	***	***	***	***
TMT			-	***	***	***	***
WHT				-	ns	ns	ns
NHCM					-	ns	ns
MHPF						-	ns
EHPF							-

*ns* not significant; *P < 0.05; **P < 0.01; ***P < 0.001

Finally, none of the nine feline sera from the C2 (NCR) group reacted to any of the food extracts tested.

### Canine inhibition ELISA

The percentage inhibition of the three meat positive inhibitor on CMT, DMT and TMT plates correlated positively with the increasing concentrations of inhibitor used; the inhibition with the negative control (BMT) also increased proportionally, due to nonspecific binding. At this concentration, the inhibition of the negative inhibitor background was minimal (<13%), whilst a strong inhibition (>50%) was present with the three-meat positive inhibitor. Consequently, we present herein the results of ELISAs obtained after incubation of the canine sera with 50 μg of the various inhibitors (50 μL of the serum + inhibitor mixtures), that is using a 1000 μg/mL inhibitor solution.

**Fig. 3:**
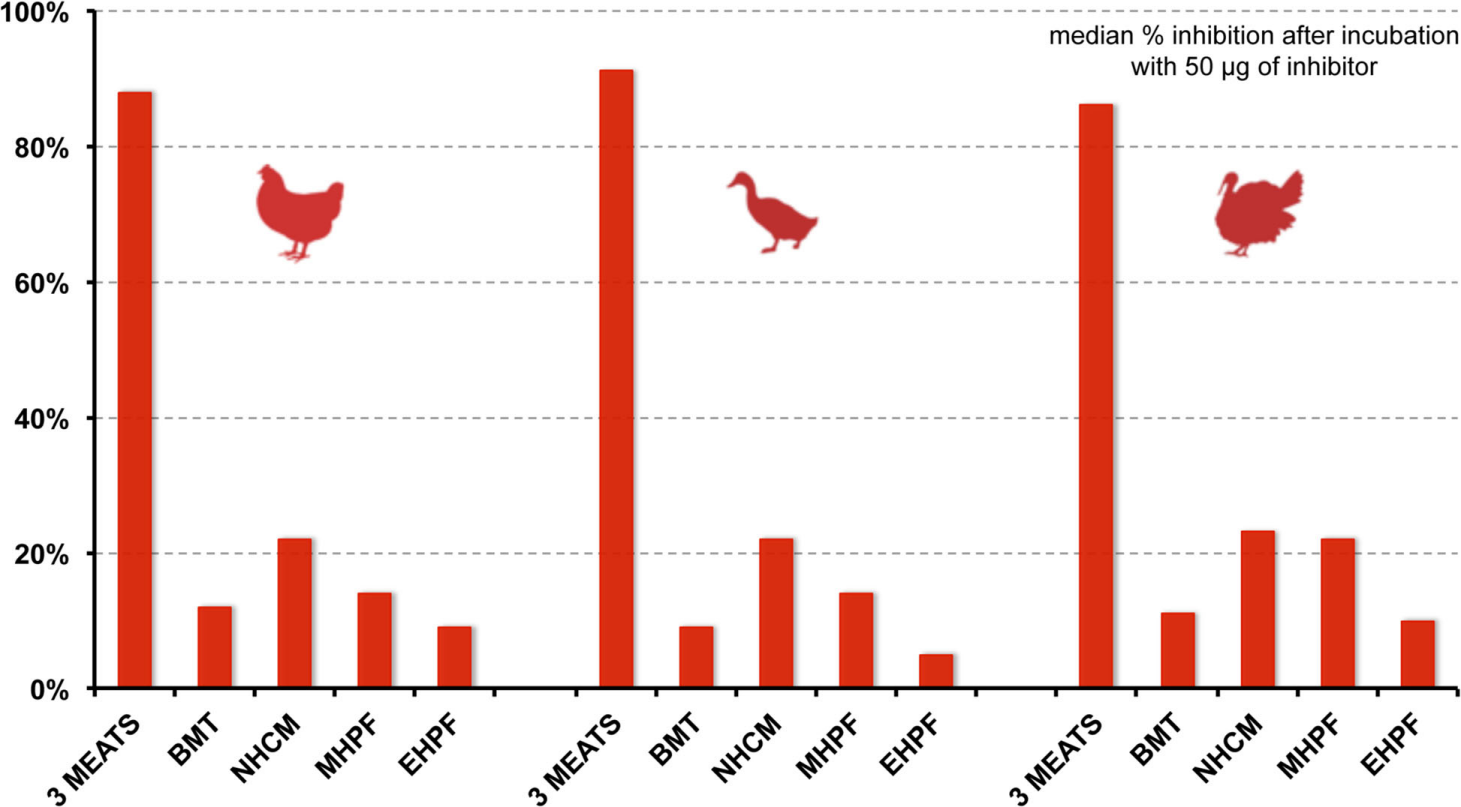
Percentages of inhibition of the reactivities with the different extracts. The ELISA coats are represented as an animal icon, while the nature of inhibitors is indicated in the x-axis. The data presented on the figure correspond to the percentages of inhibition with a 1000 μg/mL solution of the various inhibitors

The percentage inhibition with the positive control (three meats) varied between 86 and 91% depending upon the extract on which it was tested (Fig. 3); that with the negative control was between 9 and 12%. The inhibitions with the NHCM extract were ~22% of the original (no-inhibitor) reactivities, those of the MHPF varied between 14 and 22%. Finally, incubation with the EHPF led to inhibitions of only 5 to 10%, which were always lower than those obtained with the negative inhibitor BMT.

### Immunoblotting

**Fig. 4:**
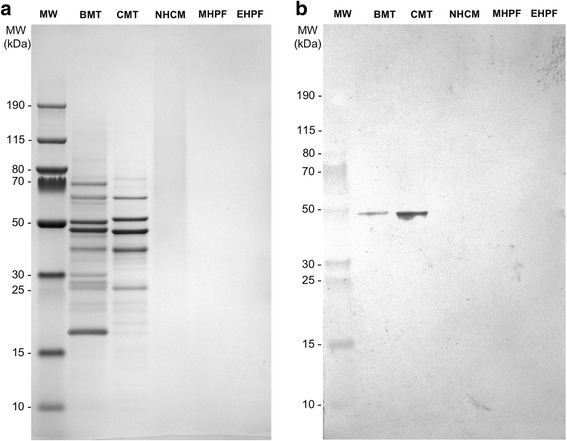
SDS-PAGE (**a**) and immunoblotting (**b**). **a**: Extracts (5 μg/lane) were separated in 4–12% gels by SDS-PAGE. **b**: Western immunoblotting of the same extracts. The blot was incubated with a chicken-reactive dog serum pool at a 1:10 dilution and revealed with anti-dog IgE-AP at 0.5 μg/mL followed by NBT/BCIP revelation. After incubation with this dog serum pool, a 48 kDa protein band was found to be the target of IgE in the CMT and BMT extracts; bands were not visible with the other extracts, however

Sodium dodecyl sulfate polyacrylamide gel electrophoresis was used to separate the extract proteins under reduced and denatured conditions. Six distinct bands of approximately 17, 42, 48, 51, 62 and 69 kDa molecular weight were visible in the BMT extract (Fig. 4a lane 2). Similarly, five bands of approximately 26, 40, 48, 52 and 62 kDa molecular weight were observed in the CMT extract (Fig. 4a lane 3). There were no detectable protein bands in the 10 to 190 kDa gel separation range in the NHCM, MHPF and EHPF extracts.

Western blot analysis was then performed to evaluate the presence of IgE binding proteins in the same extracts. Canine chicken-reacting sera contained IgE that recognized only one protein of approximately 48 kDa molecular weigth in the CMT extract (Fig. 4b, lane 3). Interestingly and surprisingly, a likely similar 48 kDa band, albeit weaker in intensity, was also found in the BMT control extract. In contrast and as expected, there were no visible bands in the NHCM, MHPF and EHPF extracts.

## Discussion

With these experiments, we provide evidence that only the extensive, but not the partial, hydrolyzation of poultry feathers prevents their recognition by serum IgE from dogs and cats sensitized to poultry. Furthermore, we found the EHPF to be unable to inhibit any of the IgE reactivity to chicken, duck or turkey meats, thereby suggesting that such hydrolysate could be of value as a restrictive commercial diet, even in dogs or cats with IgE hypersensitivity to the meats of any of these three poultry species.

Using sera from AFR-suspected dogs and cats with undetectable, low, moderate or high levels of chicken-specific IgE, we first performed ELISAs using chicken, duck or turkey meat extracts. As could be expected from the results of a recent serological study [10], between 84 and 97% of these sera not only had IgE that recognized chicken meat, but they also identified those of duck and turkey. The lower frequency of positivity to duck and turkey was only seen in the group (LCR) that had the lowest reactivity to chicken.

As these three meat extracts come from taxonomically related species, the co-reactivity we observed likely represents true allergenic cross-reactivity rather than improbably frequent co-sensitizations. Further ELISA inhibition studies with poultry meats and eggs should be performed to expand on the newly-proven cross-reactivity that exists between commonly eaten mammalian meats [8, 10]. Our chicken-sensitized canine and feline sera also recognized—albeit with a lower prevalence of reactivity—an extract made from a chicken meal (NHCM). This observation is not surprising as this chicken carcass-derived product usually contains small amounts of chicken flesh.

To confirm these ELISA results, SDS-PAGE and immunoblotting were performed. Electrophoresis confirmed the lack of detectable bands in the NHCM, MHPF and EHPF extracts, which suggests that residual proteins, if any, would likely be below the lowest resolution allowed by this gel, that is 10 kDa. Immunoblotting done with a pool of three dogs with high chicken-specific IgE confirmed the lack of 10 to 190 kDa proteins targeted by IgE in the NHCM, MHPF and EHPF extracts.

Interestingly, immunoblotting revealed that IgE from the chicken-reactive canine sera identified a single 48 kDa in the chicken meat, and likely the same protein in the beef meat extract. While the identity of this protein remains unknown, a likely candidate is Gal d 9, the chicken muscle beta-enolase, a newly-characterized chicken allergen for food allergic humans [15]. Whilst this allergen was found recently to cross-react with fish enolase in some patients with the “chicken-fish” food allergy syndrome [15], it also has an 86% identity with the bovine beta-enolase (ENO3) and is remarkably conserved across species. The presence of a band of similar molecular weight in the BMT and CMT extracts suggests the possibility of some cross-reactivity existing between these two allergen sources, a hypothesis supported by the common co-sensitization found between beef and chicken in our recent serological survey [10]. Our ELISA inhibition studies do not support an extensive cross-reactivity, however, as there was only minimal inhibition of the CMT IgE reactivity by the BMT extract. Further studies should be performed to characterize this 48 kDa chicken allergen and determine its cross-reactivity potential with meats from other mammalian, poultry and fish species.

In this study, there was low-to-absent IgE recognition of the two tested poultry feather extracts, and the frequency of positive tests depended upon the degree of hydrolysis of the product. At first, we hypothesized that poultry feathers, which are different in protein composition from that of meat, would not be recognized by IgE from chicken-sensitized companion animals. While this suspicion was verified with the feline sera, we found that serum IgE from 40% of the dogs identified the partially-hydrolyzed extract. Furthermore, our IgE ELISA inhibition studies confirmed that the MHPF extract did block between 14 to 22% of the original reactivity to the chicken, duck or turkey meats. These results imply the existence of some residual allergen cross-reactivity between poultry meats and feathers. In fact, the “bird-egg syndrome” of humans is a well-known example of such cross-reactivity [16]). In these patients, there is secondary chicken meat and egg yolk allergy due to a primary IgE sensitization to bird feathers with IgE recognition of chicken serum albumin (also known as alpha-livetin or Gal d 5) as an allergen [17]. Whether or not dogs with IgE recognizing the MHPF have IgE against Gal d 5 is unknown, but this is deserving of further study. The presence of residual albumin at the feather’s quill’s inferior umbilicus is anatomically plausible and quite probable, and it provides an argument against feathers being entirely “novel” protein sources.

In contrast to the observations above, the extensively-digested poultry feather extract was not identified by IgE from any of our canine and feline sera, even by those with the highest levels of chicken-specific IgE. Furthermore, the percentage of inhibition of the original reactivity to chicken, duck or turkey meats was lower than that of the negative beef meat inhibitor. These results suggest that the extensive hydrolyzation of poultry feathers leads to a complete or near-complete lack of IgE recognition, even in pets with high levels of serum poultry IgE. Our ELISA inhibition data also revealed the apparent absence of allergenic cross-reactivity of this EHPF with any of the three poultry types of meat tested. These serological testing results suggests the “anallergenicity” of this extract, and they are supported by the lack of clinical reactivity seen in ten chicken-allergic dogs that ate the EHPF-containing RCA/Ultamino commercial diet for two weeks [12].

## Conclusions

In summary, these ELISA studies suggest that it is only with an extensive hydrolyzation that a protein source is not recognized by serum IgE from sensitized animals, even in those with high levels of sensitization. As a result, commercial foods containing extensive hydrolysates, such as the RCA/Ultamino, would be valuable to diagnose AFRs in dogs and cats, as shown by our recent trial of ten chicken-allergic dogs challenged negatively with this diet [12]. Alternatively, they might be of interest as the sole food source for animals with high levels of sensitization, even to the parent proteins of similar species. One should not forget, however, that AFR in dogs might be also associated with non-IgE immune mechanisms, for example, those due to T-lymphocyte activation [18–22]. Whether or not extensively-hydrolyzed diets would be valuable in such animals has not yet been determined.

## Additional files

Additional file 1: Details of selected animal sera. (DOCX 128 kb)

Additional file 2: Positive test frequencies of canine and feline serum groups. (DOCX 74 kb)

## Abbreviations

AFR: Adverse food reaction
BMT: Beef meat
CAFR: Cutaneous adverse food reaction
CMT: Chicken meat
DMT: Duck meat
EHPF: Extensively-hydrolysed poultry feathers
HCR: High chicken reactive sera
LCR: Low-chicken reactive sera
MCR: Medium chicken-reactive sera
MHPF: Mildly-hydrolysed poultry feathers
NCR: Chicken non-reactive sera
NHCM: Non-hydrolysed chicken meal
OD: Optical density
TMT: Turkey meat
WHT: Wheat
