# Supplementary material for: Extensive protein hydrolyzation is indispensable to prevent IgE-mediated poultry allergen recognition in dogs and cats
Source: BMC Vet Res. 2017 Aug 17;13:251. doi: 10.1186/s12917-017-1183-4 (PMC5561598; doi:10.1186/s12917-017-1183-4)
Supplement: Supplementary file 1 — Details of selected animal sera. (DOCX 128 kb) [file 12917_2017_1183_MOESM1_ESM.docx]

**Additional File 1: details of selected animal sera**

*Dogs*

Pruritus was the most commonly reported presenting complaint in the selected dogs (26/40 dogs; 65%). Others were gastrointestinal signs (diarrhoea and/or vomiting, 7/40; 18%) and otitis (7/40; 18%), unspecified dermatological problems (3/40; 8%), erythema (2/40; 5%), and folliculitis (1/40; 3%).

D1 group (30 dogs in three subgroups of ten):

- D1-LCR: the ages of the dogs ranged from 1 to 9 years. Five of them were females (three neutered); two of the five males were castrated. Two were German Shepherd dogs, one dog was crossbred and the remainder were from a variety of breeds.

- D1-MCR: ages ranged between 1 to 8 years. Five of the dogs were females (three neutered); four of the five males were castrated. Two dogs were Jack Russell terriers, one was crossbred, and the others were from a variety of breeds.

- D1-HCR: the ages varied between 1 to 9 years. Five of the dogs were females (four were neutered) and four of the five males were castrated. Two dogs were crossbred; the remainder represented a mixture of different breeds.

D2-NCR group:

In this cohort, the dogs’ ages ranged from 1 to 6 years. While five were females (four neutered), four of the five males were castrated. Three dogs in this group were Labradors, with the remaining dogs being of different other breeds.

*Cats*

Dermatitis was the most frequent complaint in these cats (24/40 cats; 60%), followed by gastrointestinal signs (11/40; 28%), pruritus (5/40; 13%) and otitis (1/40; 3%).

C1 group (31 cats in three subgroups of 11, 10 and 10, respectively)

- C1-LCR: ages ranged from 1 to 9 years. Females represented six of the cats (five were neutered); four of the five males were castrated. All but one were domestic shorthaired (DSH) while the last was a Birman.

- C1-MCR: ages varied between 1 to 9 years. Four of the cats were females (three neutered); four of the six males were castrated. All the cats were DSH.

- C1-HCR: these cats aged from less than 1 to 9 years. Three of the cats were females (two were neutered); six of the seven males were castrated. One was a British shorthaired cat while all others were DSH.

C2-NCR group:

In this group of 9 cats, ages ranged from 9 months to 8 years. Six of these cats were females (five were neutered); all of the three males were castrated. One was a Persian crossbred, one was a Ragdoll, one was a Siamese and one was a domestic long hair; the other six cats were DSH.
