# Supplementary material for: Extensive protein hydrolyzation is indispensable to prevent IgE-mediated poultry allergen recognition in dogs and cats
Source: BMC Vet Res. 2017 Aug 17;13:251. doi: 10.1186/s12917-017-1183-4 (PMC5561598; doi:10.1186/s12917-017-1183-4)
Supplement: Supplementary file 2 — Positive test frequencies of canine and feline serum groups. (DOCX 74 kb) [file 12917_2017_1183_MOESM2_ESM.docx]

**Additional File 2:**

**Positive test frequencies of canine serum groups**

|  | Group D1 (%) | | |  | Group D2 (%) |
| --- | --- | --- | --- | --- | --- |
| Extracts | LCR | MCR | HCR |  | NCR |
| CMT (chicken meat) | 100 | 100 | 100 |  | 0 |
| DMT (duck meat) | 90 | 100 | 100 |  | 0 |
| TMT (turkey meat) | 90 | 100 | 100 |  | 0 |
| NHCM (non-hydrolysed chicken meal) | 40 | 90 | 90 |  | 0 |
| MHPF (mildly-hydrolysed poultry feathers) | 30 | 30 | 50 |  | 10 |
| EHPF (extensively-hydrolysed poultry feathers) | 0 | 0 | 0 |  | 0 |
| BMT (beef meet) | 0 | 0 | 10 |  | 0 |

**Positive test frequencies of feline serum groups**

|  | Group C1 (%) | | |  | Group C2 (%) |
| --- | --- | --- | --- | --- | --- |
| Extracts | LCR | MCR | HCR |  | NCR |
| CMT (chicken meat) | 100 | 100 | 100 |  | 0 |
| DMT (duck meat) | 55 | 100 | 100 |  | 0 |
| TMT (turkey meat) | 91 | 100 | 100 |  | 0 |
| NHCM (non-hydrolysed chicken meal) | 0 | 10 | 10 |  | 0 |
| MHPF (mildly-hydrolysed poultry feathers) | 0 | 0 | 0 |  | 0 |
| EHPF (extensively-hydrolysed poultry feathers) | 0 | 0 | 0 |  | 0 |
| WHT (wheat) | 9 | 0 | 10 |  | 0 |
